# Glycine N-methyltransferase deficiency in female mice impairs insulin signaling and promotes gluconeogenesis by modulating the PI3K/Akt pathway in the liver

**DOI:** 10.1186/s12929-016-0278-8

**Published:** 2016-10-04

**Authors:** Yi-Jen Liao, Tzong-Shyuan Lee, Yuh-Ching Twu, Shih-Ming Hsu, Ching-Ping Yang, Chung-Kwe Wang, Yu-Chih Liang, Yi-Ming Arthur Chen

**Affiliations:** 1 School of Medical Laboratory Science and Biotechnology, College of Medical Science and Technology, Taipei Medical University, Taipei, Taiwan; 2 Department and Institute of Physiology, National Yang-Ming University, Taipei, Taiwan; 3 Department of Biotechnology and Laboratory Science in Medicine, School of Biomedical Science and Engineering, National Yang-Ming University, Taipei, Taiwan; 4 Department of Biomedical Imaging and Radiological Sciences, National Yang-Ming University, Taipei, Taiwan; 5 Department of International Medicine, Taipei City Hospital Ranai Branch, Taipei, Taiwan; 6 Department of Microbiology, College of Medicine, Kaohsiung Medical University, Kaohsiung, Taiwan

**Keywords:** Glycine N-methyltransferase, Triglycerides, Insulin signaling, PI3K/Akt pathway, Liver

## Abstract

**Background:**

Glycine N-methyltransferase (GNMT) is abundantly expressed in the normal liver but is down-regulated in liver cancer tissues. GNMT knockout (Gnmt−/−) mice can spontaneously develop chronic hepatitis, fatty liver, and liver cancer. We previously demonstrated that hepatic GNMT is decreased in high-fat-diet-induced type 2 diabetes mellitus, but its contribution to metabolic syndrome is unclear. Here we show that GNMT modulates key aspects of metabolic syndrome in mice.

**Methods:**

Eleven-week-old Gnmt−/− and wild-type (WT) mice with a C57BL/6 genetic background were used in this study. The metabolic defects of GNMT deficiency were measured by glucose and insulin tolerance tests, lipid homeostasis, gluconeogenesis, and insulin signaling.

**Results:**

Gnmt−/− mice, especially females, exhibited glucose intolerance and insulin resistance. However, their body fat and lean mass, food and water intakes, and energy expenditure did not differ from those of WT mice. In addition, glucose-stimulated insulin secretion and insulin-stimulated glucagon secretion were normal in the serum and pancreatic islets of Gnmt−/− mice. Importantly, we found that GNMT deficiency increased lipogenesis and triglycerides in the liver. The elevated triglycerides disrupted the ability of insulin to induce Akt and S6 ribosomal protein phosphorylation, and then triggered insulin resistance and gluconeogenesis in female Gnmt−/− mice.

**Conclusions:**

Our data indicate that hepatic GNMT regulates lipid and glucose homeostasis, and provide insight into the development of insulin resistance through modulating the PI3K/Akt pathway.

**Electronic supplementary material:**

The online version of this article (doi:10.1186/s12929-016-0278-8) contains supplementary material, which is available to authorized users.

## Background

Metabolic syndrome is a constellation of interrelated disorders that include type 2 diabetes mellitus (T2DM), insulin resistance, dyslipidemia, fatty liver, and atherosclerosis [1]. The pathogenesis of metabolic syndrome is multifactorial, but lipids, glucose, and inflammation manifested as insulin resistance appear to be crucial features [2]. T2DM has reached epidemic proportions worldwide [3]. The rapid increase in the prevalence of T2DM in recent decades is due to the interaction of genetic susceptibility and environmental factors such as inappropriate diet and sedentary lifestyles [4–6]. Insulin resistance is correlated with dyslipidemia and nonalcoholic fatty liver disease [7], which is the most common type of liver disease worldwide.

Glycine N-methyltransferase (GNMT) catalyzes the synthesis of sarcosine from glycine using S-adenosylmethionine (SAM) as the methyl donor, and plays an important role in the regulation of the hepatic SAM pool [8]. GNMT also functions as a folate-binding protein and cytosolic receptor for clearing environmental carcinogens by regulating hepatic detoxification pathways [9, 10]. There is increasing evidence that GNMT plays a crucial role in the pathophysiological features of liver diseases, including chronic hepatitis, glycogen storage, hypercholesterolemia, fatty nodules, and liver cancer [11–15]. In addition, a lack of SAM (in a methyl-deficient diet) also causes liver cancer and steatohepatitis in rodents [16, 17]. A particularly important finding in both our own studies and those of others is that hepatic GNMT is down-regulated in dietary models (e.g., methionine/choline-deficient, high-cholesterol, and high-fat diets) of induced T2DM but not in genetic model (e.g., ob/ob mice) [18, 19]. Hepatic GNMT is reported elevated in streptozotocin-treated rats [20], a missense mutation (*fatty, fa*) in the leptin receptor gene (ZFD) rats [21], and retinoic acid/dexamethasone-treated rats [22], suggesting that the regulatory mechanisms of GNMT in the liver differ between type 1 diabetes and T2DM, and between dietary and genetic models. Since GNMT is also found in pancreatic tissue [23], whether GNMT is involved in the regulation of insulin signaling and T2DM is largely unknown.

In this study we investigated the role of hepatic GNMT in insulin signaling and the underlying molecular mechanisms. Genetic deletion of GNMT impaired glucose tolerance and insulin sensitivity via the accumulation of triglycerides and deregulation of insulin-stimulated Akt activation and gluconeogenesis in the liver.

## Methods

### Animals and diet

Eleven-week-old wild-type (WT) and GNMT knockout (Gnmt−/−) mice [11, 12] with a C57BL/6 genetic background were used in this study. All mice were maintained on standard chow (5001, LabDiet, St Louis, MO, USA) and housed in a 12-/12-h light/dark cycle. The experimental protocols were approved by the Institutional Animal Care and Use Committee of Taipei Medical University.

### Glucose and insulin tolerance tests

For glucose-tolerance tests, the mice (*n* = 8–10 per group) were fasted overnight for 16 h. After measuring the fasted blood glucose level, each mouse received intraperitoneal (i.p.) injection of 20 % glucose at 2 g/kg body weight (Delta Select, Dreieich, Germany). Blood glucose levels were then measured after 15, 30, 60, 90, and 120 min. For insulin-tolerance tests, the blood glucose levels were measured after mice had fasted for 3 h. Each mouse received an i.p. insulin injection at 0.75 U/kg body weight (Actrapid, Novo Nordisk, Bagsvaerd, Denmark), and the blood glucose levels were then measured after 15, 30, 45, and 60 min.

### Physiological metabolic analyses

The metabolic rate, food and water intakes and body fat and lean mass of WT and Gnmt−/− mice (*n* = 3 or 4 per group) were analyzed using the LabMaster Calorimetry Module (TSE Systems, Bad Homburg, Germany), Tecniplast^®^ device, and Skyscan 1076 device, respectively.

### Glucagon and insulin secretion

All animals (*n* = 4–7 per group) received an i.p. injection with insulin (100 IU/ml, Actrapid, Novo Nordisk, Bagsvaerd, Denmark) at 0.75 U/kg body weight or 20 % glucose at 2 g/kg body weight. After 20 min, the pancreatic and blood samples were collected for immunohistochemistry (IHC) staining and ELISA analyses.

### IHC and ELISA

Paraffin-embedded pancreatic sections were incubated with the antibodies against glucagon (1:200 dilution, Lot No. #8233) and insulin (1:100 dilution, Lot No. #4590) (Cell Signaling, Beverly, MA, USA) and detected using the Universal LSAB^TM2^ kit (DakoCytomation Carpinteria, CA, USA) according to the manufacturer’s instructions. The serum insulin levels were measured using mouse insulin ELISA kit (Cat. #EZRMI-13 K, Millipore, Billerica, MA) in accordance with the manufacturer’s instructions. The whole blood samples were directly drawn into a centrifuge tube without anti-coagulant. Let blood clot at room temperature for 30 min. Centrifuge the clotted blood at 2,000 to 3,000 × g for 15 min at 4 °C and the serum samples were subjected to insulin quantification.

### Western blotting

Liver tissues were lysed using a lysis buffer supplemented with protease and phosphatase inhibitors [24]. Proteins (50 μg) were separated using an 8-12 % SDS gel. The following antibodies (1:1000 dilution) used in this study were purchased from Cell Signaling: phospho-Akt (Ser-473, Lot No. #4060), total-Akt (Lot No. #4691), phospho-S6 ribosomal protein (Ser240/244, Lot No. #4858; and Ser235/236, Lot No. #5364) and total-S6 ribosomal protein (Lot No.#2317), and phospho-insulin receptor β (IR-β, Tyr1150/1151, Lot No. #3024). Total-IR-β (Lot No. sc-711) was purchased from Santa Cruz Biotechnology. Immunoblotting signals were normalized to those for α-tubulin (1:5000 dilution, Lot No. T9026, Sigma-Aldrich), and quantified by densitometric scanning.

### Gene expression analysis

Total RNA was isolated from mouse liver using TRIzol Reagent (Ambion, Carlsbad, CA, USA), according to the manufacturer’s protocol. Complementary DNA was produced from RNA (2 μg) using a SuperScript II RNase H-Reverse Transcriptase Kit (Invitrogen, Carlsbad, CA, USA). The real-time PCR reactions using SYBR Green (Applied Biosystems, Foster City, CA, USA) were assayed using an Applied Biosystems Prism 7700 sequence detection system. The specific primer sequences are listed in Additional file 1: Table S1. Predicted cycle threshold (CT) values were exported into Microsoft Excel worksheets for analysis. Comparative CT methods were used to determine the differences in gene expression relative to GAPDH. Relative amounts of mRNA level in Gnmt−/− mice were determined by calculating the ratio relative to the WT mice separately for each sex.

### Statistical analysis

Statistical analyses were performed using SPSS version 13 (SPSS, Chicago, IL, USA). Student’s *t*-test was used to compare groups. A probability value of *p* < 0.05 was considered to indicate statistical significance.

## Results

### GNMT deficiency impaired glucose tolerance, especially in female mice

**Fig. 1:**
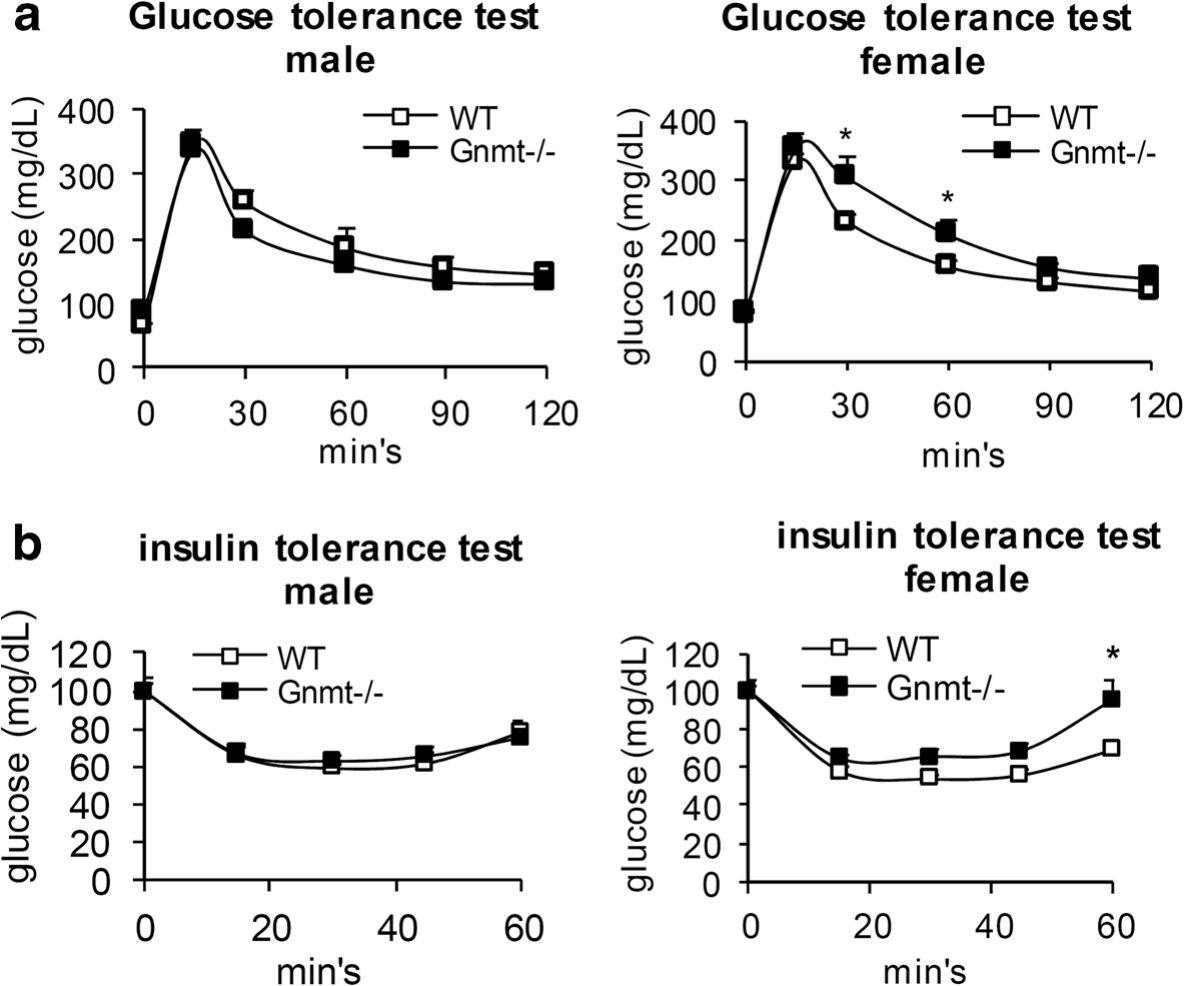
Impaired glucose metabolism in female Gnmt−/− mice. **a** Glucose tolerance testing of Gnmt−/− mice and their WT littermate controls (*n* = 8–10 per group). **b** Insulin tolerance testing of Gnmt−/− mice and their WT littermate controls (*n* = 8–10 per group). All error bars indicate s.e.m. *p < 0.05 (between WT and Gnmt−/− mice)

To investigate whether GNMT deficiency contributes to the metabolic phenotype arising from glucose intolerance and insulin resistance, we applied a glucose tolerance test and an insulin tolerance test to a Gnmt−/− mouse model. As shown in Fig. 1a, we detected an impaired glucose tolerance in female Gnmt−/− mice at 30 and 60 min after glucose administration. On the other hand, GNMT deficiency also impaired insulin sensitivity in female mice (Fig. 1b). In contrast, the findings of both glucose tolerance and insulin sensitivity tests did not differ between male Gnmt−/− mice and their WT littermate control mice (Fig. 1). These data demonstrate that compared to WT mice, female Gnmt−/− mice was more glucose intolerant and insulin resistant than male Gnmt−/− mice.

### GNMT deficiency did not impair the metabolic effects

**Fig. 2:**
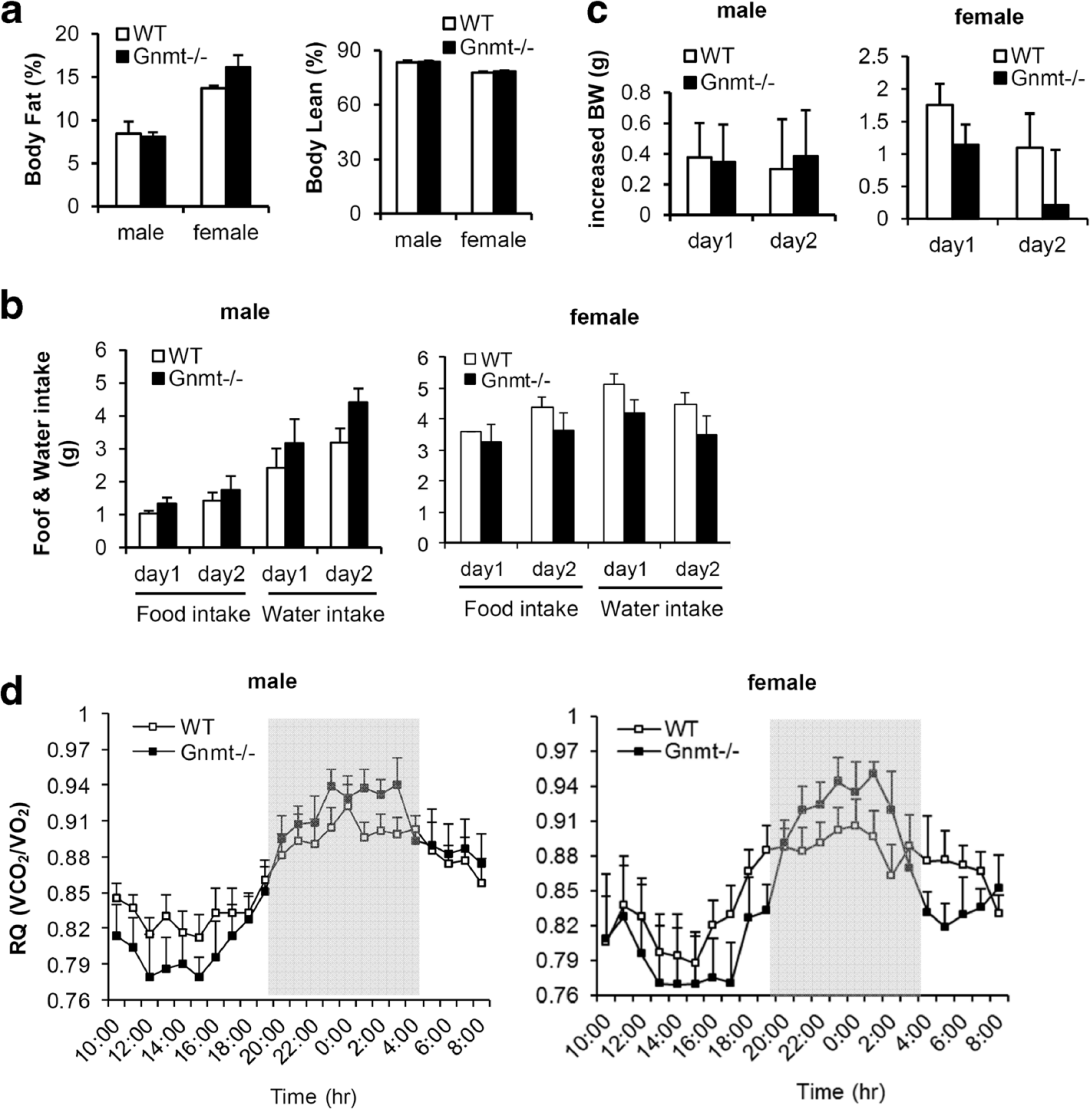
GNMT deficiency did not impair the metabolic effects. **a** Body fat and lean mass, **b** food and water intakes, and **c** increased body weight (BW) of WT and Gnmt−/− mice over 2 days (n = 3 or 4 per group). **d** Respiratory quotient (RQ) in WT and Gnmt−/− mice over 24 h (*n* = 3 or 4 per group). All error bars indicate s.e.m

Considering the effects of GNMT on controlling energy homeostasis, we first analyzed the body composition of Gnmt−/− mice. The body fat and body lean mass did not differ between Gnmt−/− and WT mice (Fig. 2a). Alterations in the basal metabolic rate were compared by carrying out a metabolic characterization in Gnmt−/− mice. The daily food and water intakes did not differ between Gnmt−/− and WT mice of the same sex (Fig. 2b). The daily increase in body weight was the same in Gnmt−/− and WT mice (Fig. 2c). Calorimetric experiments were conducted during the fed state. The respiratory quotient did not differ between Gnmt−/− and WT mice (Fig. 2d).

### GNMT deficiency did not impair the secretion function of pancreatic islets

**Fig. 3:**
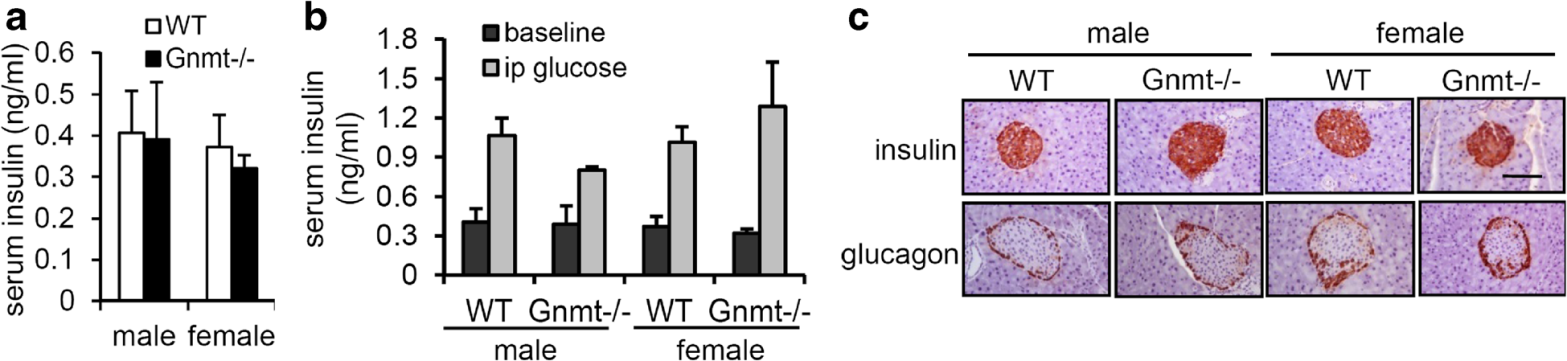
GNMT deficiency did not impair the secretion function of pancreatic islets. **a** Serum insulin levels in overnight-fasted WT and Gnmt−/− mice (*n* = 5 per group). **b** Serum insulin levels after glucose injection in WT and Gnmt−/− mice mice (*n* = 4–7 per group). **c** Insulin and glucagon staining of pancreatic islets in WT and Gnmt−/− mice. Scale bars indicate 100 μm. All error bars indicate s.e.m

To obtain a better understanding of the impact of the GNMT gene knockout on pancreatic function, we first measured the insulin levels in WT and Gnmt−/− mice. The fasting serum insulin levels did not differ between Gnmt−/− mice and their WT littermate controls for either sex (Fig. 3a). We then analyzed glucose-stimulated insulin secretion and insulin-stimulated glucagon secretion in pancreatic islets. As shown in Fig. 2b and c, glucose treatment induced normal insulin secretion. In addition, insulin-stimulated glucagon secretion in α-cell islets exhibited no alterations between Gnmt−/− and WT mice of either sex (Fig. 3c). These data suggest that impaired glucose metabolism in Gnmt−/− mice arises primarily from hepatic insulin resistance rather than impaired insulin secretion due to β-cell dysfunction.

### Increased lipogenesis and accumulated triglycerides in the liver of Gnmt−/− mice

**Fig. 4:**
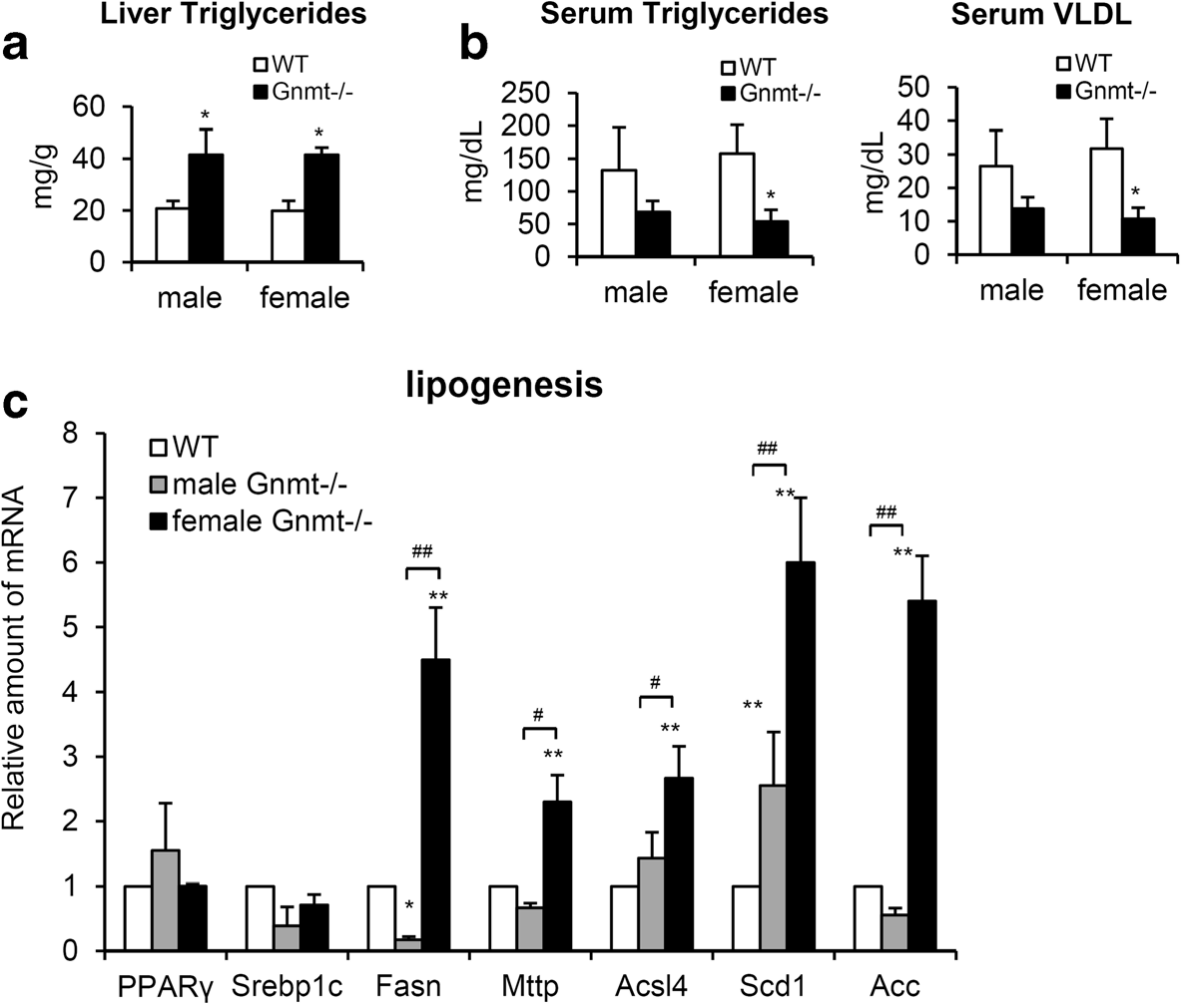
Gnmt−/− mice developed hepatic steatosis and increased de novo lipogenesis in the liver. **a** Hepatic and **b** serum levels of triglycerides and VLDL in 11-week-old WT and Gnmt−/− mice (*n* = 6 per group). **c**. Expression of genes that encode lipogenetic transcription factors (PPARγ and Srebp1c) and enzymes (Fasn, Mttp, Acsl4, Scd1 and Acc) in the liver of Gnmt−/− mice (*n* = 4 per group). * *p* < 0.05 (between WT and Gnmt−/− mice); ** *p* < 0.01 (between WT and Gnmt−/− mice); ^#^ *p* < 0.05 (between male and female Gnmt−/− mice); ^##^ *p* < 0.01 (between male and female Gnmt−/− mice)

The accumulation of hepatic triglycerides is strongly associated with insulin resistance [7, 25]. Since we found impaired systemic insulin sensitivity in female Gnmt−/− mice (Fig. 1), we subsequently evaluated the hepatic steatosis and de novo lipogenesis in these mice. Steatosis was quantified by measuring the total hepatic triglycerides level, which revealed a two-fold increase in Gnmt−/− mice relative to WT mice (Fig. 4a). In addition, the serum levels of triglycerides and very low density lipoprotein (VLDL) were both decreased in Gnmt−/− mice (Fig. 4b). Increased de novo lipogenesis and decreased VLDL secretion are crucial for the development of hepatic steatosis [25], and so we measured the expression of transcription factors that promote hepatic lipogenesis as well as genes that encode enzymes that contribute to lipogenesis. The levels of peroxisome proliferator-activated receptor-γ (PPARγ) and sterol-regulatory element-binding protein 1c (Srebp1c), which are two key regulators of lipogenetic genes in response to insulin, did not differ between WT and Gnmt−/− mice (Fig. 4c). On the other hand, fatty acid synthase (Fasn), microsomal triglyceride transfer protein (Mttp), long-chain fatty-acid-CoA ligase-4 (Ascl4), stearoyl-CoA desaturase 1 (Scd1), and Acetyl-CoA carboxylase (Acc), which are genes involved in de novo lipogenesis, were significantly zelevated in female Gnmt−/− mice relative to WT mice (Fig. 4c). Importantly, those genes involved in de novo lipogenesis were also significantly elevated in female Gnmt−/− mice relative to male Gnmt−/− mice (Fig. 4c). These data imply that the hepatic insulin resistance observed in female Gnmt−/− mice may due to increased de novo lipogenesis.

### Dysregulation of insulin-stimulated Akt activation in the liver of female Gnmt−/− mice

**Fig. 5:**
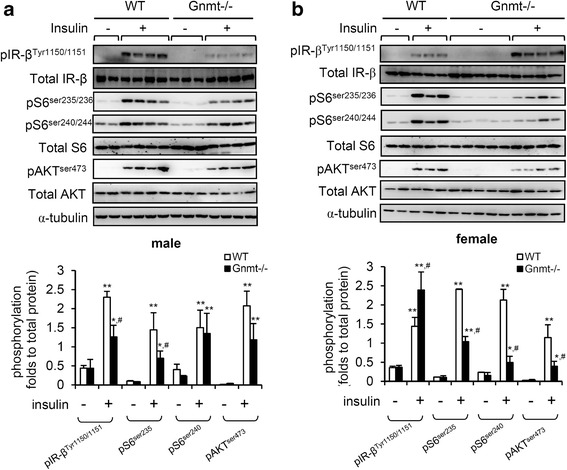
GNMT deficiency impaired insulin-stimulated Akt and S6 ribosomal protein activation in the liver. Representative Western blot analysis and quantification of expression and insulin-stimulated phosphorylation of Akt, S6 ribosomal protein, and IR in the liver of male **a** and female **b** WT and Gnmt−/− mice (*n* = 4–5 per group). α-Tubulin was used as a loading control. The levels of immunoreactive phosphorylated proteins were normalized to the total expression of the respective protein. All error bars indicate s.e.m. ** *p* < 0.01 (between baseline and insulin treatment); * *p* < 0.05 (between baseline and insulin treatment); ^#^ *p* < 0.05 (between insulin-treated WT and Gnmt−/− mice)

To further investigate the molecular basis of insulin resistance in Gnmt−/− mice, we analyzed insulin-stimulated signaling in the liver. The i.p. injection of insulin induced the phosphorylation of the IR-β, S6 ribosomal protein and Akt in WT and Gnmt−/− mice (Fig. 5). Importantly, the activation level of S6 ribosomal protein and Akt was much lower in female Gnmt−/− mice than in WT mice (Fig. 5b). The phosphorylation of IR-β was enhanced in insulin-stimulated WT and Gnmt−/− mice, however, female Gnmt−/− mice expressed more significance phosphorylation of IR-β (Fig. 5b). While, the male Gnmt−/− mice showed less phosphorylation of IR-β (Fig. 5a).

### GNMT knockdown is associated with increased expression of liver gluconeogenesis genes in female mice

**Fig. 6:**
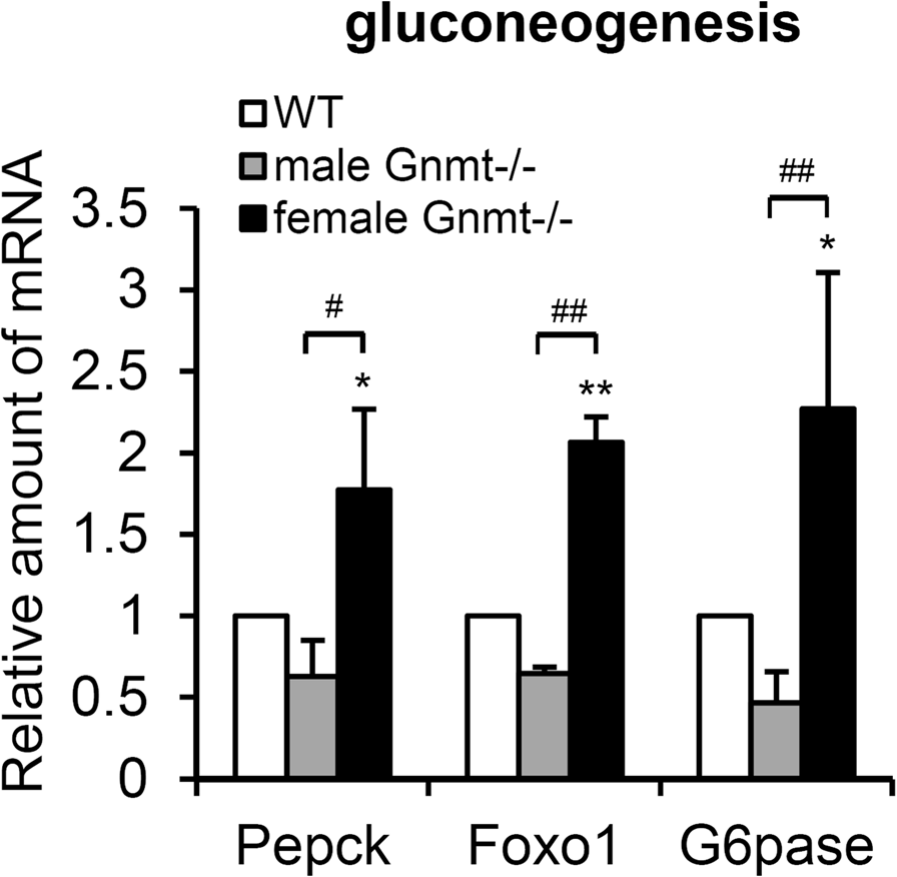
Genes involved in gluconeogenesis are upregulated in female Gnmt−/− mice. Real-time PCR analyses of genes involved in gluconeogenesis (Pepck, Foxo1, and G6pase) in the liver of Gnmt−/− mice (*n* = 4 per group). * *p* < 0.05 (between WT and Gnmt−/− mice); ** *p* < 0.01 (between WT and Gnmt−/− mice); # *p* < 0.05 (between male and female Gnmt−/− mice); ## *p* < 0.01 (between male and female Gnmt−/− mice)

To assess the mechanism underlying the impairment of whole body glucose homeostasis in female Gnmt−/− mice (Fig. 1), we analyzed the expression of genes involved in gluconeogenesis. Consistent with the physiological data obtained in the glucose tolerance test (Fig. 1) and signaling data (Fig. 5b), female Gnmt−/− mice displayed significant elevations of gluconeogenic markers (Pepck, Foxo1, and G6pase) in the liver relative to male Gnmt−/− mice (Fig. 6).

## Discussion and conclusion

**Fig. 7:**
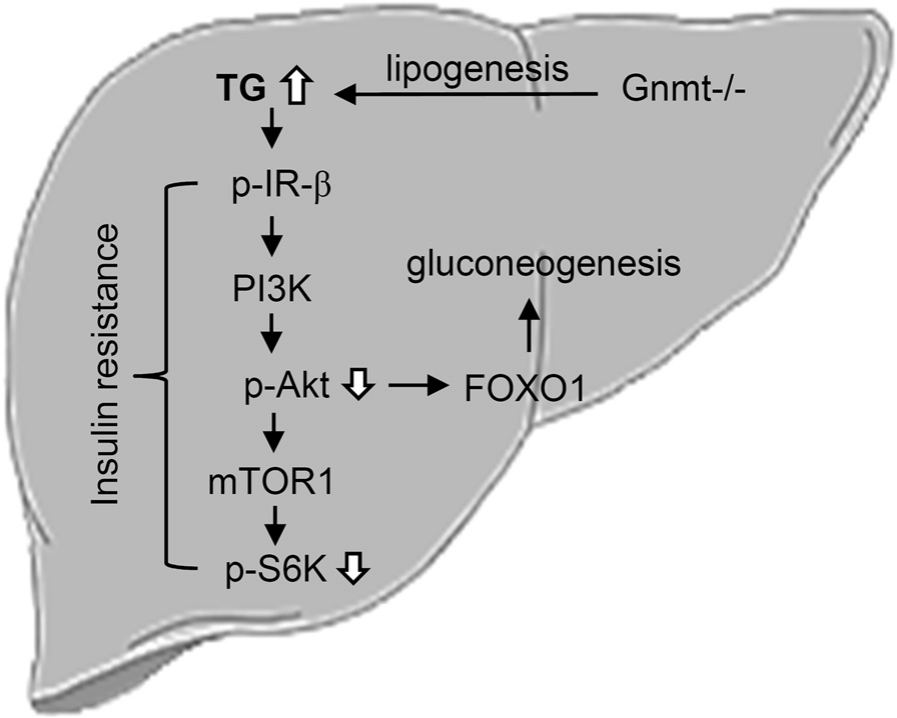
Illustration of proposed mechanism underlying GNMT deficiency induced triglycerides accumulation and insulin resistance in the liver. GNMT deficiency may enhance lipogenesis to cause hepatic triglycerides accumulation, thus impairing insulin signaling and gluconeogenesis, especially in female Gnmt−/− mice

GNMT is abundantly expressed in normal livers, but is down-regulated in high fat diet-induced T2DM and liver cancer tissues [13, 19]. GNMT deficiency in mice results in chronic hepatitis, fatty liver, and liver cancer spontaneously [11, 12, 15]. However, the molecular mechanisms underlying GNMT-deficiency-induced hepatic triglycerides accumulation and insulin signaling remain unclear. Excess SAM has been found to increase the turnover of hepatic triglycerides stored for secretion in VLDL in Gnmt−/− mice [26]. The findings of the current study support the novel concept of GNMT deficiency enhancing lipogenesis and triglycerides accumulation, subsequently triggering insulin resistance and gluconeogenesis by modulating the PI3K/Akt pathway in the liver (Fig. 7).

The liver plays a critical role in the systemic response to insulin, controlling the metabolism of both glucose and lipids [27, 28]. Hepatic lipogenesis includes the de novo synthesis of fatty acids from acetyl-CoA and malonyl-CoA and further processing into triglycerides. The produced triglycerides are then either stored in lipid droplets or packed into VLDL and exported into the blood [28]. In our study we found that the increased de novo lipogenesis and inhibition of hepatic triglycerides exported via VLDL contribute to lipid accumulation in the liver of Gnmt−/− mice (Fig. 4). Triglycerides, which are the main lipids stored in the liver of nonalcoholic fatty liver disease patients, are associated with the development of steatohepatitis and insulin resistance [7, 25, 29]. Indeed, hepatic inflammatory infiltration [19] as well as inflammatory genes (IL-6, TNF-α and IL1-β) were increased in Gnmt−/− mice (data not shown). The impairment of PI3K/Akt activation is involved in the triglycerides accumulation induced hepatic insulin resistance [7, 30]. Our study has revealed impaired insulin-stimulated Akt phosphorylation in Gnmt−/− mice (Fig. 5).

Our previous studies suggested that the rate of tumorigenesis in HCC was significantly higher in female Gnmt−/− mice [10-12, 31]. Moreover, the retinoids that induce the activation of GNMT are sex-specific [32]. In the present study we observed that female Gnmt−/− mice express a glucose intolerance phenotype (Fig. 1). The activation of the PI3K/Akt pathway is responsible for glucose metabolism, including glycogen synthesis and gluconeogenesis [33]. We found that the gluconeogenesis genes (Pepck, Foxo1, and G6pase) were up-regulated only in female Gnmt−/− mice (Fig. 6). Foxo1 stimulates hepatic gluconeogenic gene expression and is directly phosphorylated and inhibited by Akt [34, 35]. Indeed, the level of insulin-stimulated Akt phosphorylation was lower in female Gnmt−/− mice (Fig. 5b). In addition, a twofold increase in hepatic triglycerides was observed in Gnmt−/− mice of either sex, but the lipogenesis and gluconeogenesis were elevated in female Gnmt−/− mice. Although the detailed underlying mechanism is unclear, this discrepancy may due to different roles of estrogen and androgen in Gnmt−/− mice.

Previously, we reported that GNMT regulates liver cancer growth in part via interacting with the DEP domain containing mTOR-interacting protein (DEPTOR) and modulating the mTOR signaling pathway [24]. DEPTOR is an mTOR inhibitor that interacts with mTOR directly. Overexpression of DEPTOR activates Akt via the inhibition of a negative feedback loop from S6 to PI3K [36]. The phosphorylation of IR-β is able to recruit and phosphorylate IR substrate proteins after ligand binding, leading to activation of the PI3K/Akt pathway [37, 38]. However, the difference of insulin-stimulated pIR-β was observed between male and female Gnmt−/− mice (Fig. 5). Although the detailed underlying mechanism is unclear, this sex discrepancy may due to different feedback controls of hepatic glucose metabolism [12], methylation [11] or hormone [32] in Gnmt−/− mice.

GNMT is expressed predominantly in the liver, with moderate levels in proximal kidney tubules and exocrine tissue of the pancreas [8, 23]. However, GNMT deficiency does not affect the insulin and glucagon secretion of pancreatic islets (Fig. 3), implying that the dysregulation of glucose metabolism in Gnmt−/− mice may arise primarily from hepatic insulin resistance rather than impaired insulin secretion due to β-cell dysfunction. In addition to the liver, skeletal muscle and adipocytes are the main insulin-responsive tissues and are associated with the pathogenesis of T2DM [30]. Although we did not detect GNMT in adipocytes using Western blotting and Q-PCR, weak expressions of GNMT in both mRNA and protein levels were observed in the skeletal soleus muscle (data not shown). Therefore, the role of GNMT in these insulin target organs is yet to be elucidated, especially in skeletal muscle.

In conclusion, our findings indicate that GNMT deficiency exacerbates lipogenesis and the accumulation of triglycerides in the liver, further impairing insulin signaling and gluconeogenesis. GNMT may therefore be a suitable therapeutic target when treating not only dyslipidemia but also broader aspects of metabolic diseases.

## Abbreviations

Acc, acetyl-CoA carboxylase; Ascl4, long-chain fatty-acid-CoA ligase-4; DEPTOR, DEP domain containing mTOR-interacting protein; Fasn, fatty acid synthase; GNMT, glycine N-methyltransferase; Gnmt−/−, GNMT knockout; IR-β, insulin receptor β; Mttp, microsomal triglyceride transfer protein; PPARγ, peroxisome proliferator-activated receptor-γ; SAM, s-adenosylmethionine; Scd1, stearoyl-CoA desaturase 1; Srebp1c, sterol-regulatory element-binding protein 1c; T2DM, type 2 diabetes mellitus; WT, wild-type.

## Additional file

Additional file 1: Table S1. Real-time PCR primers used in the study. (DOC 38 kb)
