# Supplementary material for: Glycine N-methyltransferase deficiency in female mice impairs insulin signaling and promotes gluconeogenesis by modulating the PI3K/Akt pathway in the liver
Source: J Biomed Sci. 2016 Oct 4;23:69. doi: 10.1186/s12929-016-0278-8 (PMC5050923; doi:10.1186/s12929-016-0278-8)
Supplement: Additional file 1: Table S1. — Real-time PCR primers used in the study. (DOC 38 kb) [file 12929_2016_278_MOESM1_ESM.doc]

**Supplementary Table S1. Real-time PCR primers used in the study.**

| **Gene Name** | **Forward (5’-3’)** | **Reverse (5’-3’)** |
| --- | --- | --- |
| PPARγ | GCTCAAGTATGGTGTCCATGAGATC | TGAGATGAGGACTCCATCTTTATTCA |
| Srebp1c | GGAGCCATGGATTGCACATT | GCTTCCAGAGAGGAGGCCAG |
| Fasn | CCGTGTGACCGCCATCTATAT | AGCGGCTCGTTGTCACATC |
| Mttp | CGTCCACATACAGCCTTGAC | CCACCTGACTACCATGAAGC |
| Acsl4 | GAAAACTTGAGCGTTCCTCCAA | ACGTTCACACTGGCCTGTCA |
| Scd1 | TGGAAATGCCTTTGAGATGG | CCAGCCAGCCTCTTGACTAT |
| Acc | CCAGGCCATGTTGAGACGCT | ATCACAGAGCGGACGCCATC |
| Pepck | CTGGCACCTCAGTGAAGACA | TCGATGCCTTCCCAGTAAAC |
| Foxo1 | ACATTTCGTCCTCGAACCAGCTCA | ATTTCAGACAGACTGGGCAGCGTA |
| G6pase | TCTTGTGGTTGGGATACTGG | AGCAATGCCTGACAAGACTC |
| GAPDH | TCACCACCATGGAGAAGGC | GCTAAGCAGTTGGTGGTGCA |

GAPDH were used as an internal gene for mouse tissues.
